# Purification and characterization of an isoflavones conjugate hydrolyzing β-glucosidase (ICHG) from *Cyamopsis tetragonoloba* (guar)

**DOI:** 10.1016/j.bbrep.2019.100669

**Published:** 2019-08-08

**Authors:** Vidushi Asati, Pankaj Kumar Sharma

**Affiliations:** Department of Biological Sciences, Birla Institute of Technology and Science, Pilani, India

**Keywords:** *Cyamopsis tetragonoloba*, β-glucosidase, Legume, Isoflavonoid Glycosides

## Abstract

A β-glucosidase with high specific activity towards isoflavone glycosidic conjugates was purified from seeds of Guar (*Cyamopsis tetragonoloba*) by ammonium sulphate precipitation followed by size exclusion and ion exchange chromatography. The pH and temperature optima of the purified Isoflavones conjugate hydrolyzing β-glucosidase (ICHG) were found to be pH 4.5 and 37 °C, respectively. The enzyme was relatively stable at higher temperatures. Effect of different divalent metal ions was studied and it was found that Cobalt and Mercury ions completely inhibited the enzyme activity. K_m_ and V_max_ of the purified isoflavones conjugates hydrolyzing β-glucosidases (ICHG) was 0.86 mM and 6.6 IU/mg respectively. The enzyme was most likely a trimer (approximate Mr 150 kDa) with potential subunits of 50 kDa. The purified enzyme showed activity against isoflavone conjugate glycosides viz daidzin and genistin but was inactive towards other flavonoid conjugates. The product conversion was confirmed by HPTLC and HRMS analysis. The MALDI-TOF analysis of the ICHG showed a score greater than 78 with 20 matches in MASCOT software. The five resultant peptides obtained had highest similarity in sequence with β-glucosidase from C*icer arietinum*. The β-glucosidase from the *C. arietinum* has also been reported to exhibit the isoflavone conjugate hydrolyzing properties thus confirming the nature of the enzyme purified from the Guar seeds.

## Highlights

• A specific Isoflavones conjugate hydrolyzing β-glucosidase (ICHG) from *Cyamopsis tetragonoloba* (guar) was purified and characterized for the first time.
• The purified enzyme is characterized as a trimer with a molecular weight of 150 kDa
• The isolated enzyme can be further used in the bioengineering of the edible pods for the human consumption.

## 1 Introduction

Flavonoids and isoflavonoids represent one of the largest and most studied classes of plant based secondary metabolites with an estimate of more than 10,000 different compounds. These compounds although don't directly participate in the essential life processes, such as growth, respiration, storage and reproduction but they greatly help plants in adapting to abiotic and biotic stresses [1,2]. Flavonoids are reported to be ubiquitously present in all plants whereas isoflavonoids are indigenously present in large quantities in legumes. Recent interest in these plant based metabolites has actually kindled because of the presence of many health promoting properties such as antioxidant, anticancerous, antibacterial, antifungal etc. The functional attributes of these compounds lie in their structural properties. The core backbone consists of two phenyl groups joined by a 3 carbon bridge and then further substitution at different positions of the overall fused rings. The hydroxyl groups present at the different substitution position of the phenyl backbone further affect the activity of the enzymes which are associated with multiple pathways involved in conferring the protection to plants as well as to human beings. However, it is important to understand that these compounds many a times exist as two different forms; the one glycosylated which is water soluble, relatively inactive and preferred by plants for the storage purpose, another one is less soluble in water and the active form. These different forms of the secondary metabolites play a crucial role in the biological activities they carry out eventually; either within the plants themselves or inside the human system once consumed via food. Because of the biological activities flavonoids/isoflavonoids hold, it is extremely crucial for plants that the spatial arrangement of the metabolites within their cells is in such a way that these compounds should not hinder in normal physiological processes of the cells. Absence of such arrangement will lead to the suppression of the normal cellular processes and thus hindering the growth of the cell [3]. Different organelles of the plant cell are involved in the compartmentalization of the secondary metabolites where their precursors for the active form can be stored via certain chemical modifications. These chemical modifications include glycosylation, prenylation, methylation, acetylation etc. of the compounds which make the latter more soluble so that they are easily stored and are relatively less toxic and inactive in nature.

Glycosylation is one of the important mechanisms by which a large number of secondary metabolites get modified to be eventually stored in plant compartments which later on can be acted upon by specific hydrolytic enzymes to release their active forms. This modification is carried out mainly by the enzymes having transglycosylation activity so as to attach the sugar moieties to the aglycone molecules and thus making them suitable for storage purposes. When the plant cells undergo stress, highly specific β-glucosidases cleave the isoflavonoid/flavonoid glycosides conjugate and thus the active aglycones are released along with sugars. The aglycones then act as antioxidant, antibacterial or antifungal compounds whereas sugars are generally used for energy purposes.

β-Glucosidase belongs to the class of enzymes which mediates the hydrolysis of β-glucosidic linkages in aryl-, amino-, or alkyl-β-D-glucosides, cyanogenic glucosides, and oligo- or disaccharides. The diverse physiological roles played by this enzyme across the living system include glucoside ceramide catabolism in human tissues, cell wall, pigment and cyano-glucoside metabolism, defense against pathogens in plants, and utilization of oligosaccharide substrates by many fungi and bacteria [4]. Besides this, there are several other commercial applications for e.g. production of biofuel and ethanol from agricultural waste, hydrolysis of bitter compounds during extraction of juices, aroma liberation from wine grapes, because of which this enzyme is continuously sought after from novel resources [5]. So far the enzyme has been isolated from a vast number of living organisms ranging from lower prokaryotes to that of the higher plants and animals, both in native forms and via heterologous expression in host organisms [6]. Nevertheless, the need to look for novel sources of this enzyme from plants has always been important because of the limitations imposed by the recombinant enzymes for example glucose inhibition of the enzyme [7]. Also the identification and characterization of any enzyme from a plant source in its native form throws light on the pathways underlined in the metabolism of many putative compounds.

*Cyamopsis tetragonoloba* commonly known as guar is an edible legume which is grown in semi-arid and subtropical areas of North and North-West India predominantly in Rajasthan and East and South-East Pakistan. It is well adapted to hot climate and is drought-tolerant. Its beans are consumed as food in household and dry forage is used as livestock feed. Guar gum, which is obtained from the endosperm of the guar seeds holds extreme importance in industries as natural thickener, bonding agent, soil stabiliser, hydrocolloid, gelling agent, natural fiber, emulsifier, flocculant and fracturing agent. Apart from the industrial applications, guar has been reported to have anti-ulcer, hypolipidemic, cell-protective, hypoglycemic, and anti-secretory bioactivities. These health beneficiary activities can be accredited to the presence of secondary metabolites of polyphenolic class such as daidzein, naringenin, caffeic acid, gallotannins, genistein, gallic acid, catechol, luteolin, chlorogenic acid, ellagic acid, myricetin-7-glucoside-3-glycoside, quercetin, catechin, 2,4,3,-trihydroxybenzoic acid, kaempferol, rutin, hydroxycinnamic acid, texasin-7-O-glucoside, and *p*-coumaroylquinic acid [[8], [9], [10], [11]]. The almost exclusive presence of isoflavonoids in legume seeds is the another key point which make them striking candidates for development as pharmaceutical and nutraceutical additives in foods. Soybean (Glycine max) and Chickpea (*Cicer arietinum*) are two of those legumes for which extensive reports of the presence of isoflavonoids/flavonoids are available [12,13]. However, metabolic studies *vis-à-vis* the same, particularly w.r.t. biotransformation of the inactive metabolites into active ones, are lacking for many of the edible plants, including guar. The specific glucosidases will cleave the conjugates bonds at specific functional groups and thus expressing the advantages over the other non-specific glucosidases. Therefore, the present study was undertaken with the view of purifying the specific β-glucosidase from seeds of the guar involved in the bioconversion of isoflavonoids glycosides conjugates to their aglycone form.

## 2 Materials and methods

### 2.1 Plant material

The dried seeds of *Cyamopsis tetragonoloba* were purchased from local vendor in Pilani belonging to Jhunjhunu district of Rajasthan. The same was verified by Prof. Jitendra Panwar (Botanist), Department of Biological Sciences, BITS, Pilani – Pilani Campus. The seeds were stored at room temperature till further use.

### 2.2 Chemical and reagents

All the reagents used in enzyme purification were procured from HiMedia Laboratories except the matries for column chromatography viz Sephadex G-75 and DEAE-Sepharose which were obtained from Sigma-Aldrich (St. Louis, MO, USA). Flavonoid and isoflavonoid standards daidzein, daidzin, genistin, genistein, hesperidin, hesperetin, naringin, naringenin, rutin hydrate and quercetin used were also obtained from Sigma-Aldrich, while the solvents hexane, toluene, chloroform, ethyl acetate, methanol, acetone, formic acid and glacial acetic acid (AR grade), from HiMedia. The TLC plates (precoated with aluminum-backed silica gel 60 F_254_) were procured from Merck (Darmstadt, Germany).

### 2.3 Enzyme purification

All the experiments described below were carried out at 0–4 °C unless otherwise specified.

#### 2.3.1 Crude extract preparation

200 g of powder of dried seeds of *Cyamopsis tetragonoloba* was extracted with 800 ml of chilled 0.1 M sodium phosphate buffer pH 6 in a beaker with continuous stirring for 30 min. Crude preparation was collected after centrifugation at 10,000 rpm for 30 min at 4 °C. 1% w/v of polyvinylpolypyrrolidone (PVPP) was added to this crude extract and kept for stirring for 3 h. Role of PVPP is to remove phenolic compounds present in the seeds so as to decrease interference with the enzyme activity. The extract was centrifuged again for 10 min at 10,000 rpm. Supernatant was then collected and further subjected to ammonium sulphate precipitation.

#### 2.3.2 Ammonium sulphate precipitation

The crude extract was slowly brought to 30% ammonium sulphate saturation and was stirred for 3 h. The suspension was centrifuged at 10,000 rpm for 30 min. Supernatant was collected and then further brought down to 80% ammonium sulphate saturation. Pellet was collected after centrifugation at 10,000 rpm for 30 min. The pellet obtained was dissolved in minimum amount of extraction buffer containing 0.02 M sodium phosphate buffer (pH 6) and then subjected to size exclusion chromatography.

#### 2.3.3 Size exclusion chromatography

The dissolved pellet was loaded on to Sephadex G-75 column (1.8 cm × 50 cm) pre-equilibrated with 0.02 M sodium Phosphate buffer (pH 6). Post sample loading, 60 fractions of 2 ml each were collected at a flow rate of 1 ml/min. O. D at 280 nm was measured followed by checking for beta glucosidase enzyme activity as described later. Fractions with maximum enzyme activity were pooled for further purification.

#### 2.3.4 DEAE-Sepharose ion exchange chromatography

The pooled enzyme fractions from (c) were loaded onto DEAE-sepharose column (1.8 × 10 cm) previously equilibrated with 0.02 M sodium phosphate buffer (pH 6). The column was eluted with a linear gradient of NaCl solution from 0 to 1.0 M at a flow rate of 0.5 ml/min. Fractions of 2 ml each were collected and checked for absorbance at 280 nm followed by enzyme activity. Protein estimation at each purification step was performed using Lowry method [14].

### 2.4 Biochemical characterization of purified enzyme

#### 2.4.1 Enzyme assay

β-glucosidase activity was routinely checked at each step of purification using the synthetic substrate para nitrophenyl -β-d- glucopyranoside (PNPG). A reaction mixture of 200 μl was set up consisting of 80 μl of assay buffer (0.1 M Citrate phosphate buffer; pH 4.5), 50 μl 10 mM PNPG, 50 μl distilled water and 20 μl enzyme preparation and was incubated at 37 °C for 30 min. Reaction was terminated by adding 800 μl of 1 M Na_2_CO_3_. The absorbance was measured at 405 nm using V-630 UV–visible spectrophotometer (Jasco Corporation, Japan).

#### 2.4.2 Characterization studies

Biochemical characterization studies of the enzyme were performed using PNPG with respect to pH optima, temperature optima, metal ion dependence and enzyme kinetics. All the assays were performed in accordance with the protocol mentioned in above section with necessary changes incorporated as per the experimental requirements. To determine the pH optimum for enzyme activity, the assay buffer was prepared by varying pH from 3.5 to 10 {pH range of 3.5–6.5 (Citrate Phosphate Buffer, 0.1 M); pH 7 to 8 (Tris-HCl buffer, 0.1 M) and pH 9–10 (Carbonate-Bicarbonate buffer, 0.1 M)}. The data points were plotted to yield the pH profile for the activity. The temperature optimum of ICHG was determined by incubating the enzyme-substrate mixtures over a temperature range of 28 °C–70 °C for 30 min and terminating the reaction by adding 1 M Na_2_CO_3_. The effects of selected cations on enzyme activity were determined by adding the following metal ion salts at concentration of 5 mM final concentration: HgCl_2_, CoCl_2_, NaCl, ZnSO_4_, MnSO_4_, MgCl_2_, CaCl_2_ and CuSO_4_. Each assay buffer was prepared with the addition of these ions and enzyme-substrate mix was incubated for 30 min. For enzyme kinetics studies, PNPG was used at different substrate concentration in range of 0.1 mM–5 mM were taken and V_max_ and K_m_ were calculated using Lineweaver-Burk plot.

#### 2.4.3 Determination of molecular weight

The molecular weight of the purified enzyme was determined by size exclusion chromatography using Sephadex G-75 column with the dimensions of 1.8 × 50 cm. Elution was performed using 0.02 M sodium phosphate buffer (pH 6). The standards proteins of molecular weights ranging from 66 kDa to 124 kDa were used. The graph was plotted between log_10_ of molecular weight and the elution volume and the molecular weight of the enzyme was determined using this straight line graph.

#### 2.4.4 Sodium Dodecyl Sulphate (SDS) polyacrylamide Gel Electrophoresis (PAGE)

Proteins obtained at each step were subjected to SDS-PAGE analysis as per standard procedure with adequate modifications. Samples were denatured in 2X sample buffer containing 60 mM Tris (pH 6.8), 25% glycerol, 2% SDS, 14.4 mM 2-mercaptoethanol, 0.1% bromophenol blue and boiled for 5 min. Electrophoresis was performed on a Mini Protean® Tetra-Cell instrument (BioRad Laboratories, USA) at a constant voltage of 80 kV. After electrophoresis, the gel was stained with Coomassie Brilliant Blue G-250 stain and was observed in a BioRad Gel DocTM XR imaging system. The molecular mass of the protein bands was determined by interpolation from a semi-logarithmic plot of relative molecular mass versus the R_f_ value (relative mobility).

#### 2.4.5 Peptide mass fingerprinting

The purified ICHG band from the above step was excised from the gel and it was diced to small pieces. These pieces were further destained for 10 min intervals (3–4 times) until they became translucent white. Dehydration of gel was performed using acetonitrile and concentrated till complete dryness. Rehydration of gel was carried out with DTT followed by incubation with iodoacetamide. Two subsequent incubations with ammonium bicarbonate solution and acetonitrile for 10 min were then done and the gel was completely dried under vacuum. To this dried gel, trypsin solution was added and incubated overnight at 37 °C. The digest solution was then transferred to fresh eppendorf tubes. The gel pieces were extracted thrice with extraction buffer and the supernatant was collected each time into the single Eppendorf tube and then completely dried using Speedvac. The dried pepmix was suspended in Tris-Acetate buffer. The peptides obtained were mixed with α-Cyano-4-hydroxycinnamic acid (HCCA) matrix in 1:1 ratio and the resulting 2 μl was spotted onto the MALDI plate. After air drying the sample, it was analyzed on the MALDI TOF/TOF ULTRAFLEX III instrument and further analysis was done with FLEX ANALYSIS SOFTWARE for obtaining the peptide mass fingerprint. The masses obtained in the peptide mass fingerprint were submitted for MASCOT search in “CONCERNED” database for identification of the protein.

#### 2.4.6 Enzyme activity with natural substrates

For testing the ability of the purified β-glucosidase to hydrolyze natural isoflavonoid and flavonoid O-glycosides, commercially obtained glycoside standards viz. genistin and daidzin (isoflavonoids); rutin, naringin and hesperidin (flavonoids) were used. The assay mixture contained 155 μl Citrate Phosphate Buffer (0.1 M, pH 4.5), 25 μl isoflavonoid or flavonoid substrate (dissolved in DMSO: Citrate Phosphate buffer:1:1, 10 mM) and 20 μl of the purified enzyme. After incubating the reaction mixture for 3 h at 50 °C, reaction was terminated using 200 μl Methanol. Negative control in the form of zero-time control was used where methanol was added prior to enzyme followed by incubation as mentioned above. The reaction mixture was then centrifuged at ambient temperature for 1 min at 3000 rpm. The supernatant was collected and was subsequently extracted thrice using chloroform. The resultant extracts were completely dried and dissolved in 50 μl of absolute methanol. The enzymatic product formation was checked using High Performance Thin Layer Chromatography (HPTLC). TLC plates with 0.2 mm pre-coated silica gel 60 F_254_ (Merck, Germany) of 8 × 10 cm were taken. The enzymatic products were spotted using Linomat 5 automated sample spotter (CAMAG) using 100 μl syringe (Hamilton, Switzerland). Sample solution (2.5 μl) was spotted as bands of 6 mm width with the syringe with 5 mm distance between each spot. The spotted plate was developed in glass twin trough chamber (10 × 10 cm, CAMAG). Toluene: ethyl acetate: acetone: formic acid:20:4:2:1 was used as mobile phase. All the standard compounds dissolved individually in absolute methanol were loaded as reference. The length of the chromatogram was 80 mm from application position. After development, the TLC plate was dried using hot air oven at 50 °C and scanned densitometrically at 254 nm. The image of the developed TLC plate was captured using TLC Visualizer (CAMAG) at 254 nm. The following scan conditions were applied: Slit width: 4 × 0.10 mm, distance from Y-position: 10 mm and distance from X-position: 10 mm.

#### 2.4.7 High resolution Mass spectrometry (HRMS) analysis of the product

The analyses of the reaction mixtures were performed by HRMS on an Agilent 6545 Q-TOF LC/MS platform at positive Dual ESI ionization mode. The HRMS data were acquired at a full scan mode, with mass range of 100–1500 *m*/*z*. ESI conditions: capillary temperature 350 °C; capillary voltage 3000 V; tube lens −43.46 V. Nitrogen was used as sheath gas (30 arb) and auxiliary gas (10 arb). The products formed were analyzed using the Agilent database library MassHunter associated with the instrument. Pure standards which were commercially obtained from Sigma-Aldrich (USA) were also ran along with the samples and the spectrum was obtained for them for the comparative analysis.

## 3 Results and discussion

### 3.1 Purification of ICHG

In another report we have already shown the presence of the ICHG enzyme activity in the seeds of Guar [15]. The main aim of the current study was to further purify, characterize and sequence the ICHG so as to get a detailed information regarding its properties and to understand how much similarity there exists with other such enzymes as reported in literature. ICHG from guar seeds was purified from crude extract by means of ammonium sulphate precipitation, size exclusion chromatography and anion exchange chromatography. Protein precipitation by 30–80% ammonium sulphate proved to be a very simple but efficient step to selectively separate the unwanted proteins from the ICHG. Size exclusion chromatography was performed using Sephadex G-75 matrix which further separated proteins on the basis of their molecular weight. The presence of single band on SDS-PAGE analysis (Fig. 5) in the fraction isolated from last purification step indicates that purification of ICHG has been achieved which was also confirmed by the peptide mass fingerprinting. The low yield of the purified ICHG after final step implies that the enzyme has tendency to lose the activity easily with time. But it is important to mention that only two chromatography steps were involved to achieve the purification viz size exclusion and ion exchange chromatography as against the usage of multiple columns followed in similar studies from other legumes (Table 2).

### 3.2 pH and temperature optima

**Fig. 1:**
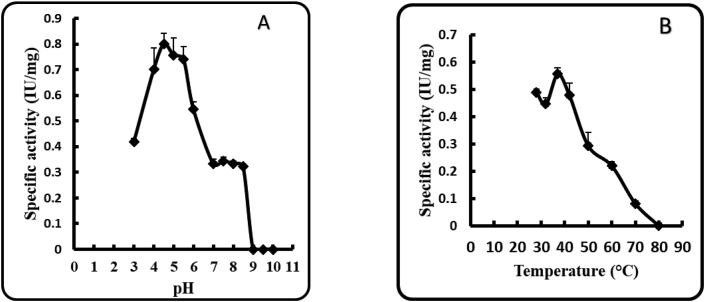
Effect of pH (A) and temperature (B) on the guar ICHG activity. For calculating pH optima, 0.1 M assay buffers from 3.5 to 10 were used {3.5 to 6.5 (Citrate Phosphate Buffer, 0.1 M); pH 7 to 8 (Tris-HCl buffer) and pH 9–10 (Carbonate-Bicarbonate buffer)}.

The biochemical characterization of the purified enzyme showed a sharp optimum pH curve with a maximum activity at pH 4.5 (Fig. 1A). which is in accordance with the preliminary studies done using the crude extract. The ICHG activity kept increasing from pH 3.0, reached maximum at 4.5 and then decreased and finally after pH 8.5, declined drastically. At pH values of 4, 5 and 5.5, around 85% of maximum activity was retained whereas at pH 6, this value came down to 62%. After this the enzyme showed very less activity in range of pH 7–8.5 but after that complete loss of activity was observed. When compared with the enzymes isolated from other plants with similar activity, certain variations were seen. The pH optima of ICHG from soybean and chickpea are 6.5 and 6 respectively [16,17]. ICHG from another non-edible legume Thai rosewood has a pH optimum of 5 [18]. This shows that the same enzyme when isolated from different sources exhibit a lot of variation in the biochemical parameters. Temperature optima for the ICHG was observed as 37 °C (Fig. 1B). A very crucial point which must be mentioned here is that at slightly higher temperatures, some activity of enzyme was still retained. As observed from Fig. 1 B, at 42 °C, 85% of maximum activity was still there. As the incubation temperature was further increased to 50 °C, 50% of activity was mainly because of the denaturation of enzyme caused by the heat. At 80 °C, total loss of activity was there. When compared with β-glucosidase from other putative legumes viz chickpea and soybean, it can be concluded that the purified enzyme exhibits a broad range of optimum temperature since in the latter mentioned plants, the maximum enzyme activity was obtained at 45 °C and 30 °C respectively [17,19]. Similarly, β-glucosidase from Thai rosewood showed temperature optima of 30 °C [18].

### 3.3 Effect of metal ions on ICHG activity

**Fig. 2:**
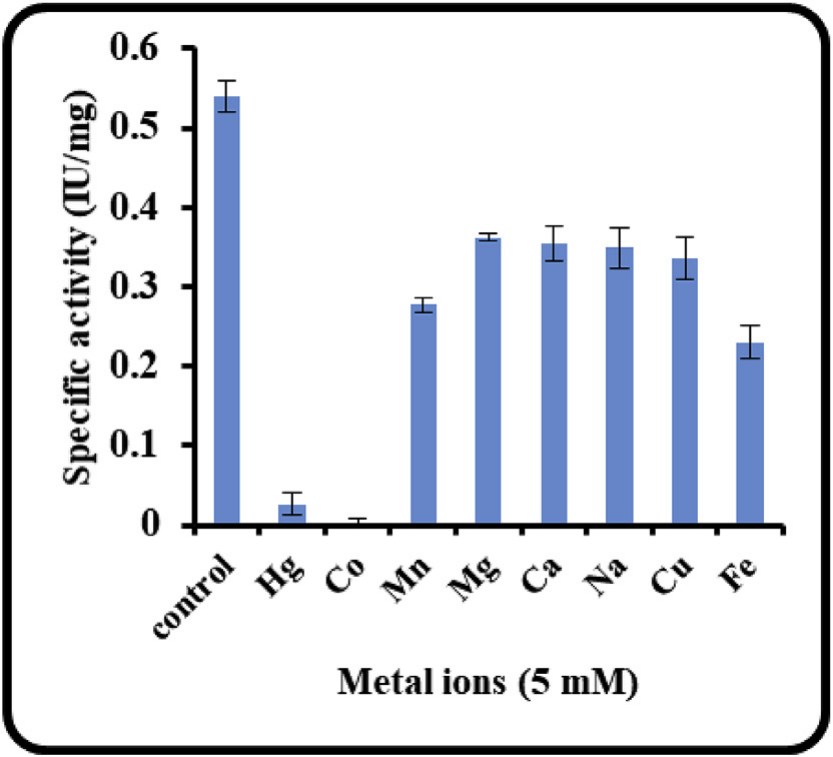
Effect of divalent metal ions on ICHG activity of seeds of Guar.

The effect of metal ions on the activity of ICHG was studied and it was observed that all the divalent metal ions affected the enzyme activity differently (Fig. 2). There was complete inhibition by divalent ions Hg^2+^ and Co^2+.^ The enzyme sensitivity to inhibition by Hg^2+^ is consistent with the reports that it interacts with the cysteine residues present in the sulfhydryl linkages, thus indicating the presence of sulfhydryl linkages in the ICHG structure [20]. Surprisingly Co^2+^ ions which did not inhibit beta glucosidase activity from other sources, showed 100% inhibition in this case. Divalent cobalt ions are known to form the complexes with several amino acids [21]. The total loss of enzyme activity in presence of the cobalt ions could indicate that the amino acids present in the active sites of the ICHG bound to cobalt ions irreversibly and hence completely denaturing its activity. Other ions like Mg^2+^, Mn^2+^, Ca^2+^, Na^+^, Cu^2+^ and Fe^3+^ also inhibited the enzyme activity albeit to moderate levels. These metals also tend to form the metallo complex with the proteins and here by altering their structure which eventually deactivate them. Inhibitory effect of these ions and np enhancement of the activity in their presence when compared to control also imply that the ICHG purified from the guar seeds is not a metal ion dependent protein.

### 3.4 Enzyme kinetics

**Fig. 3:**
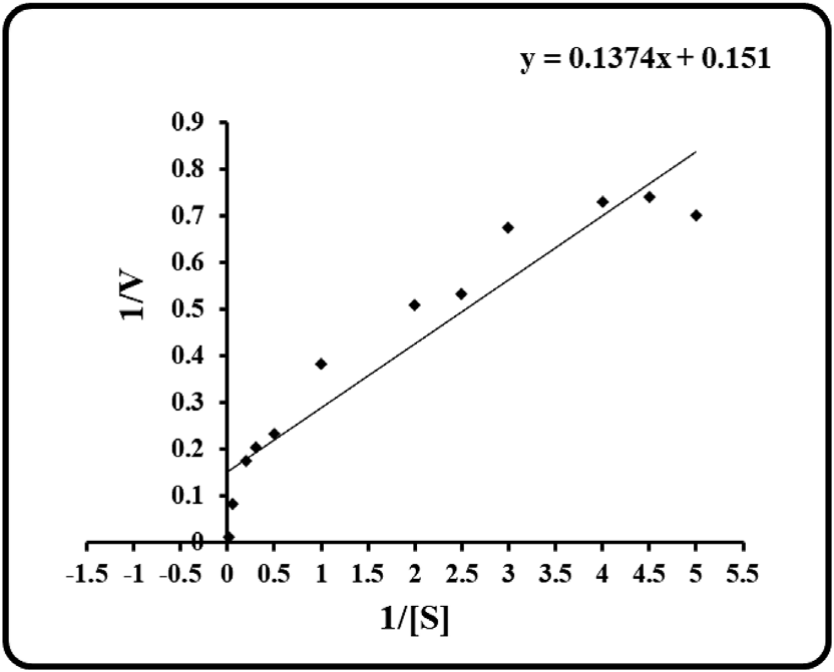
Lineweaver-Burk plot for the ICHG purified from seeds of Guar (R^2^ = 0.9031).

The *V*_max_ and *K*_m_ for β-glucosidase from the seeds of Guar were calculated using β-PNPG as substrate and carrying out the reaction at optimum pH and temperature. *V*_max_ and *K*_m_ were found to be 6.6 IU/mg glucosidase and 0.86 mM, respectively as observed from the Lineweaver-Burk plot (Fig. 3). The initial reaction rate versus substrate concentration plot for the purified β-glucosidase isolated followed Michaelis–Menten kinetics. V_max_ and K_m_ of the similar enzymes isolated from legumes like chickpea and soybean, were 18.6 IU/mg; 0.0015 mM and 20.0 IU/mg; 0.0013 mM respectively [19,21]. Relatively high value of *K*_m_ of guar enzyme denotes that it is having low affinity for the synthetic substrate. Nevertheless, the HPTLC profile where enzyme activity was carried out with natural substrates showed that the enzyme is able to cleave the isoflavonoid glycosides specifically. The kinetic studies of ICHG isolated from the soybean and chickpea involved the substrate concentration dependent assay with respect to natural compounds like daidzin and genistin.

### 3.5 Molecular weight determination

**Fig. 4:**
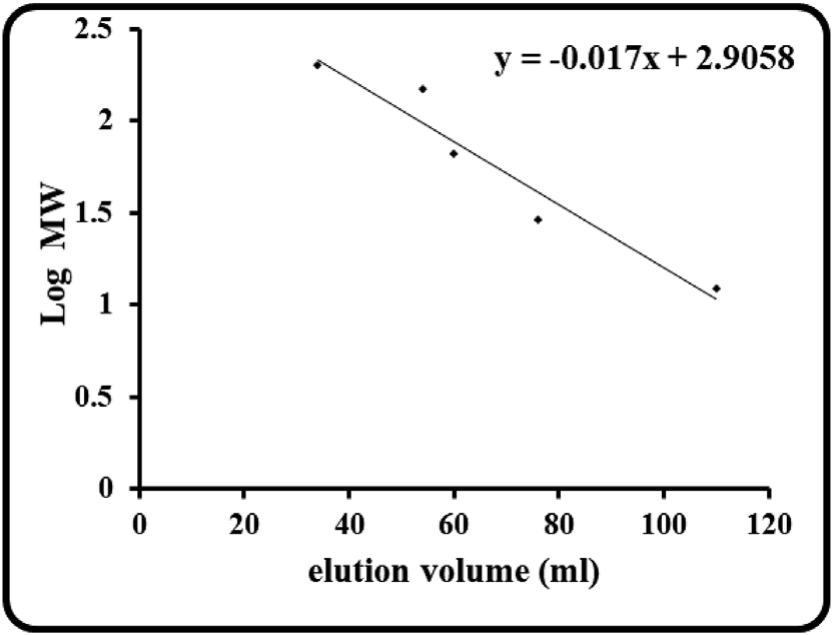
Plot of Log MW vs elution volume to calculate the molecular weight of guar ICHG. The ICHG was eluted out at 40 ml and thus the corresponding Log_10_ MW was equal to 2.17 giving a native molecular weight of ~150 kDa.

**Fig. 5:**
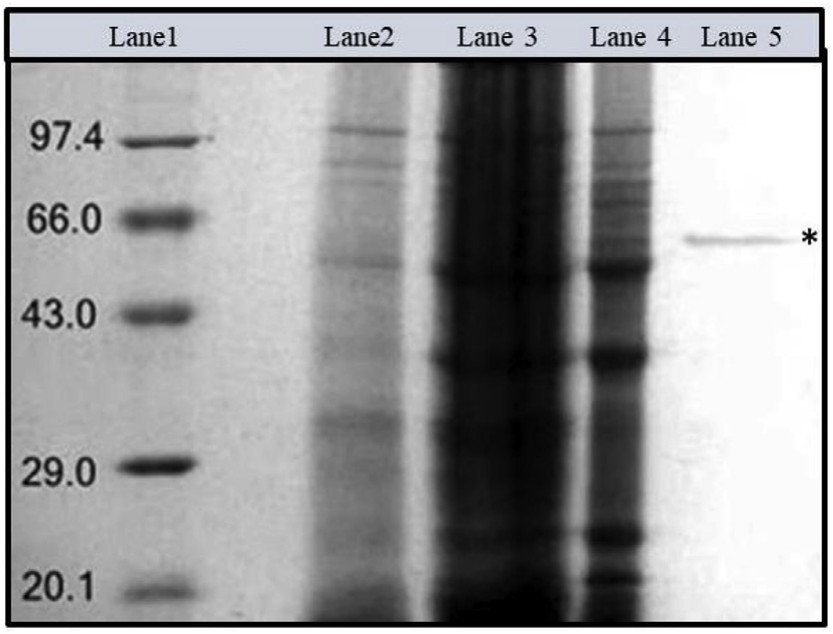
SDS-PAGE analysis of different steps in ICHG purification. Lane 1: Molecular marker. Lane 2: Crude extract. Lane 3: Ammonium Sulphate Precipitate(30–80%). Lane 4: Size exclusion chromatography fraction. Lane 5: Ion exchange chromatogrpahy fraction. *Purified ICHG enzyme.

The molecular mass of purified β-glucosidase from Guar seeds as determined by size exclusion chromatography was approximately 150 kDa (Fig. 4). Under denaturing conditions, the purified ICHG yielded a single protein band on SDS-PAGE and migrated with an apparent molecular mass of approx. 50 kDa, as determined by relative mobility (Fig. 5). The molecular mass determined by size exclusion and SDS-PAGE analysis indicated that the enzyme possibly consists of three identical subunits. Despite the relative similarity in the functions exhibited by this enzyme in different organisms, there is a huge variation in terms of the molecular weight of the same protein when purified from different sources. β-glucosidase enzyme from bacteria *Thermus flavus* has a molecular weight as low as 49 kDa [22] whereas functionally similar enzyme purified from *Aspergillus niger* had a molecular weight of 200 kDa [23]. When the purified ICHG was compared with similar ICHG from other plant sources, it was found that ICHG from soybean is homodimeric in nature with 58 kDa as its subunit [16] and ICHG from chickpea is also homodimeric with 68 kDa as its subunit [17]. β-glucosidase isolated from Thai Rosewood is that of pentameric nature having 66 kDa as the subunit weight and total molecular weight of 330 kDa.

### 3.6 Peptide mass fingerprinting

**Fig. 6:**
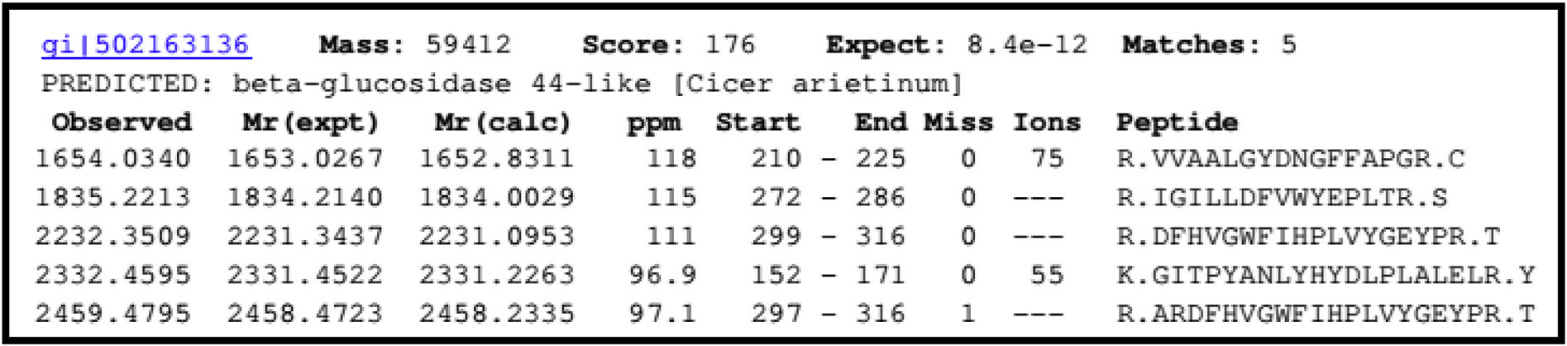
MALDI-TOF and MS analysis of purified enzyme ICHG showed maximum similarity with isoflavone-7-O-glucoside-specific beta-glucosidases from *Cicer arietinum. (Score 176)*.

The MALDI-TOF analysis of purified and denatured β-glucosidase from the Guar seeds after SDS–PAGE was done and amino acid sequences of five internal peptides were obtained whose amino acid composition is given in Fig. 6 (see Table 1). Furthermore, the sequences of the internal peptides were analyzed using MASCOT server against NCBI protein database to find out the closest β-glucosidases. The sequence matches with more than the score of 78 were considered significant matches. Around 20 matches with a MASCOT score greater than 78 were observed during analysis. Though the complete protein sequence for the guar ICHG is not available so far but considering the top twenty sequence matches from Table 2, the enzyme purified belongs to Family 1 glycosides hydrolases. Functional and structural studies for enzymes belonging to Family 1 have shown that these proteins have (α/β)_8_-barrel shape structure hereby implying that the enzyme from guar consists of this highly conserved structure at its active site. The MALDI-TOF analysis also showed sequences of the five resultant peptide sequences are closest to those of β-glucosidase from C*icer arietinum* (Chickpea). The β-glucosidase from the *C. arietinum* has also been reported to exhibit the isoflavones conjugates hydrolyzing properties [24] confirming the functional nature of the enzyme purified from the Guar seeds.

**Table 1:** Purification of Isoflavone conjugates hydrolyzing β-glucosidases from seeds of *Cyamopsis tetragonoloba*.

Fraction	Total protein (mg)	Total activity (IU)	Specific activity (IU^a^ 10^−3^/mg)	Yield (%)	Purification fold
Crude extract	3440	10.32	3.0	100	–
Ammonium sulphate	530	1.06	2.0	9.6	0.66
Sephadex G-75	256	0.768	3.0	7.4	1
DEAE-Sepharose	9.28	0.0074	0.8	0.07	0.26^a^

a Low purification yield of ICHG could be accounted for loss of significant enzyme activity over the duration of 6 days which is the time taken to complete the experiment.

**Table 2:**
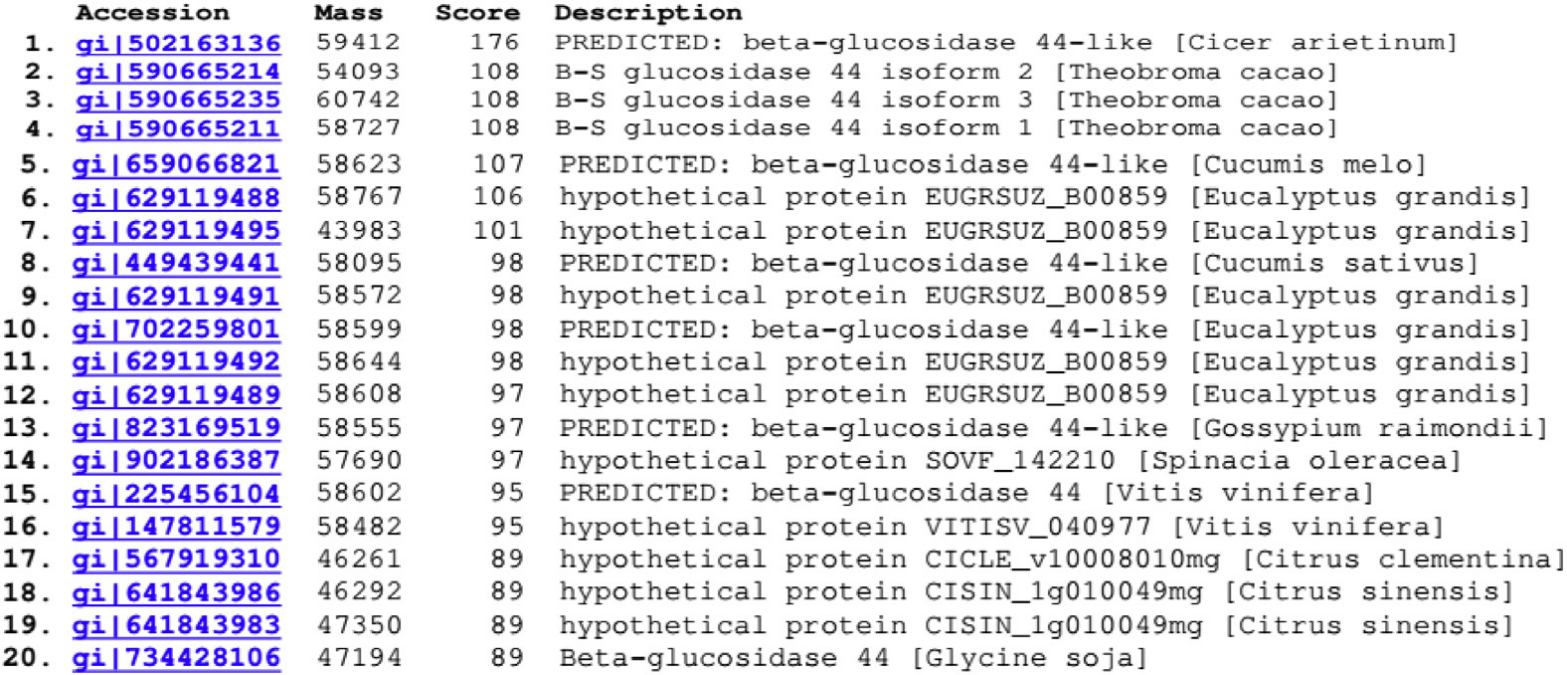
Peptide sequence matches as observed using MASCOT software.

### 3.7 Enzyme activity with natural substrates

**Fig. 7:**
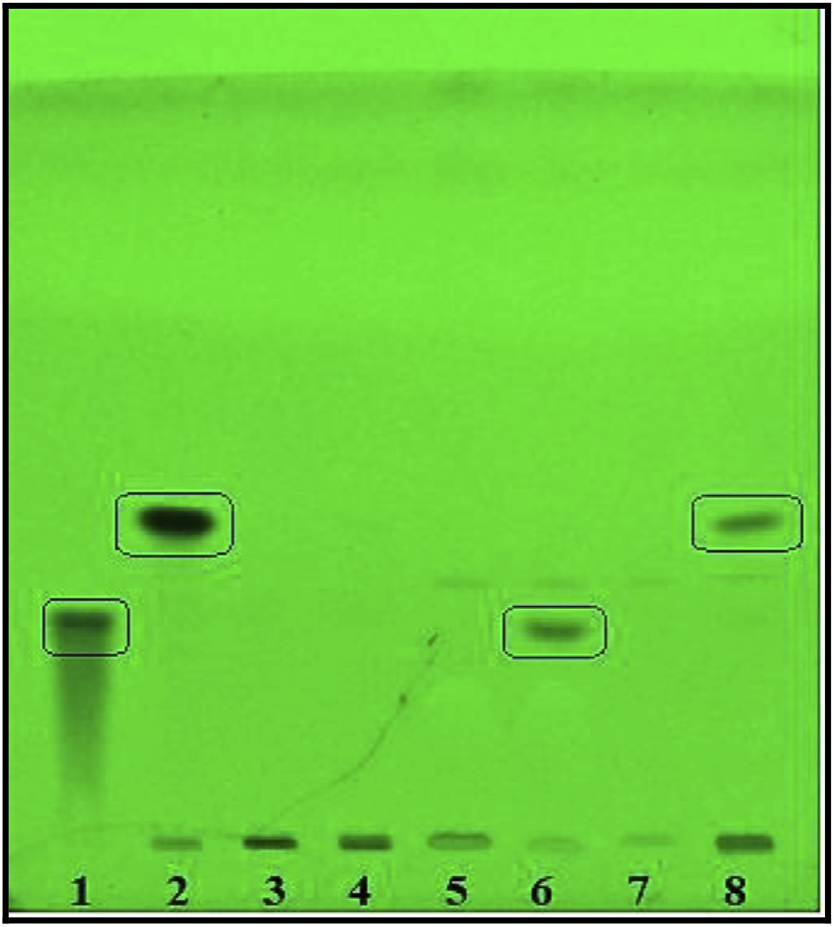
HPTLC for activity of β-glucosidase purified from Cyamopsis tetragonoloba with natural substrates. Samples loaded- 1- Daidzein standard; 2- Genistein standard; 3- Daidzin standard; 4- Genistin standard; 5- Daidzin Assay Control, 6-Daidzin Assay Test; 7-Genistin Assay Control, 8-Genistin Assay Test.

The purified ICHG purified from guar seeds was tested with different natural flavonoid and isoflavonoid glycoside substrates. The results obtained from the assay for isoflavonoid glycoside hydrolyzing β-glucosidase showed that the seeds of *Cyamopsis tetragonoloba* contain specific β-glucosidase activity towards the conjugated glycosides of isoflavonoids. The R_f_ values of the products formed, when ran on HPTLC and observed under the scanner matched with that of the corresponding standards for aglycones (daidzein; R_f_ = 0.3) and (genistein R_f_ = 0.41), respectively (Fig. 7). The enzyme activity with conjugated glycosides of flavonoid substrates like rutin, naringin and hesperedin did not show any conversion to their aglycone forms (data not shown). Thus a β-glucosidase which is highly specific towards the isoflavonoid glycosyl conjugates has been isolated from the seeds of Guar. ICHG reported in other legumes like soybean has been known to be capable of cleaving the malonylated and acylated conjugates as well. In fact, the benzenoid substituent on the condensed ring of isoflavonoid structure is present at 3-position as compared to that of 2-position in case of flavonoids. This structural difference between the isoflavonoids and other flavonoids could account for the ability of the native enzyme's active site to bind the former but not the latter [25].

### 3.8 Analysis of the enzymatic product via HRMS

To further confirm the identity of the product released upon the enzymatic reaction, HRMS was performed and the mass spectrum obtained from the product was matched against that of the standards. Fig. S1- S3 (Supplementary file) represent the HRMS spectra for the standard Daidzein, Experimental control and test for Daidzin respectively. As observed in Fig. S1, the standard daidzein showed a major peak at *m*/*z* value of 255.05. Fig. S2 represents the HRMS of the experimental control where reaction was stopped at zero time and hence the enzymatic conversion of daidzin to its aglycone daidzein was not expected. So a mass spectrum peak for the glycoside daidzin was expected at 417.186 which can be seen in Fig. S1. This is in accordance with the HPTLC results from Fig. 7 as well. The experimental test sample showed the conversion of the glycoside to aglycone as seen in Fig. 7 and hence the mass spectrum of the same should give majority of peak for daidzein. A look at Fig. S3 indicates that the product released post ionization gave *m/z* similar to that of the standard daidzein from Fig. S1 hence concluding that the resulting product from the enzymatic action of ICHG is daidzein only. Similar kind of results were obtained for the samples containing genistin as substrate where the mass spectra of product released matched with that of the pure standard as represented in Figs. S4–S6 (Supplementary file).

**Fig. 8:**
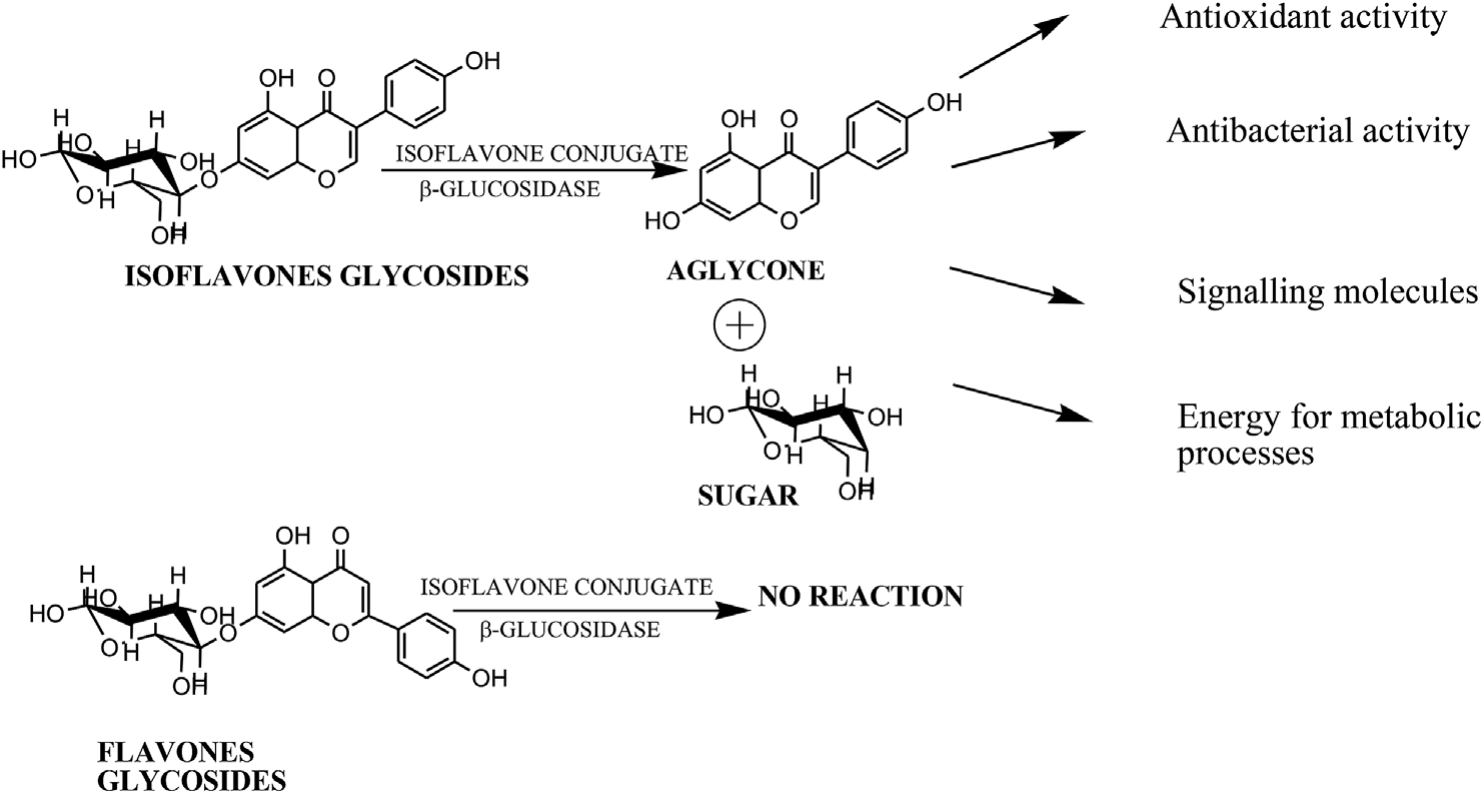
ICHG acts on Isoflavonone conjugated glycosides to release active aglcyones along with glucose.

In conclusion, this is the first time, a isoflavone specific Isoflavone conjugates hydrolyzing β-glucosidase (ICHG) from seeds of *Cyamopsis tetragonoloba* has been purified and characterized. The purified enzyme is indeed a ICHG has been confirmed by the peptide mass fingerprinting where its showed the maximum similarity with the already reported enzymes. The hydrolysis of glucosyl isoflavone conjugates is biologically relevant for plants since the aglycone part released upon the action of enzymes is more active and participates in imparting the antibacterial, antioxidant, antifungal activities. The sugar moiety freed as a by-product can be used for energy requirements (Fig. 8).

Isoflavonoids are the metabolites which are restricted mainly to the legume family of plants and since most of the legumes are consumed by the human beings, the isolation of the ICHG can be of high significance to them as well. It has been reported that when the biological activities of these compounds on human population are considered, the aglycone part of the conjugates are the one which holds health promoting properties and importance [26]. Because of the highly stable nature of the conjugated glycosylated compounds, the preparation of the food products doesn't impact much on their chemical nature and the conversion from the conjugated glycosides to aglycones occurs at very limited level. Hence, bioavailability of the isoflavones depends largely on the action of the specific glucosidases present in the human gut. This again is restricted by the inadequate presence of the specific glucosidases in our system having ICHG activity results in the non-absorption of many valuable compounds. The ICHG from Guar seeds can be a potential candidate in biotechnology industry to achieve the enzyme mediated conversion of stored glycosides conjugates of isoflavonoids to their active form. Similarly, it also opens up the possibility of using a native enzyme from this source to achieve the bioconversion of the isoflavonoid conjugates in other edible products e.g. tofu, soy milk. Several reports are there citing the biotransformation of the glycosides using the enzymes of microbial origin [27,28]. In continuation to this, ICHG purified from the guar seeds presents an interesting case where enzyme from novel source maybe used. Several attempts have been made to genetically engineer the legumes leading to an increased availability of beneficiary aglycone for the consumption of human population [29]. But such genetic manipulation cannot be achieved without the identification and characterization of ICHG activity in the concerned plant. With the ever increasing demand of enriched food in the market, the bioengineering of the edible guar pods can be explored so as to cultivate them with increased content of health promoting isoflavonoid aglycones. Such kind of research will be of high significance in terms of dealing with problems related to food for local populations.
