# Supplementary material for: Purification and characterization of an isoflavones conjugate hydrolyzing β-glucosidase (ICHG) from Cyamopsis tetragonoloba (guar)
Source: Biochem Biophys Rep. 2019 Aug 8;20:100669. doi: 10.1016/j.bbrep.2019.100669 (PMC6700428; doi:10.1016/j.bbrep.2019.100669)
Supplement: Multimedia component 2 [file mmc2.docx]

**Supplementary information**


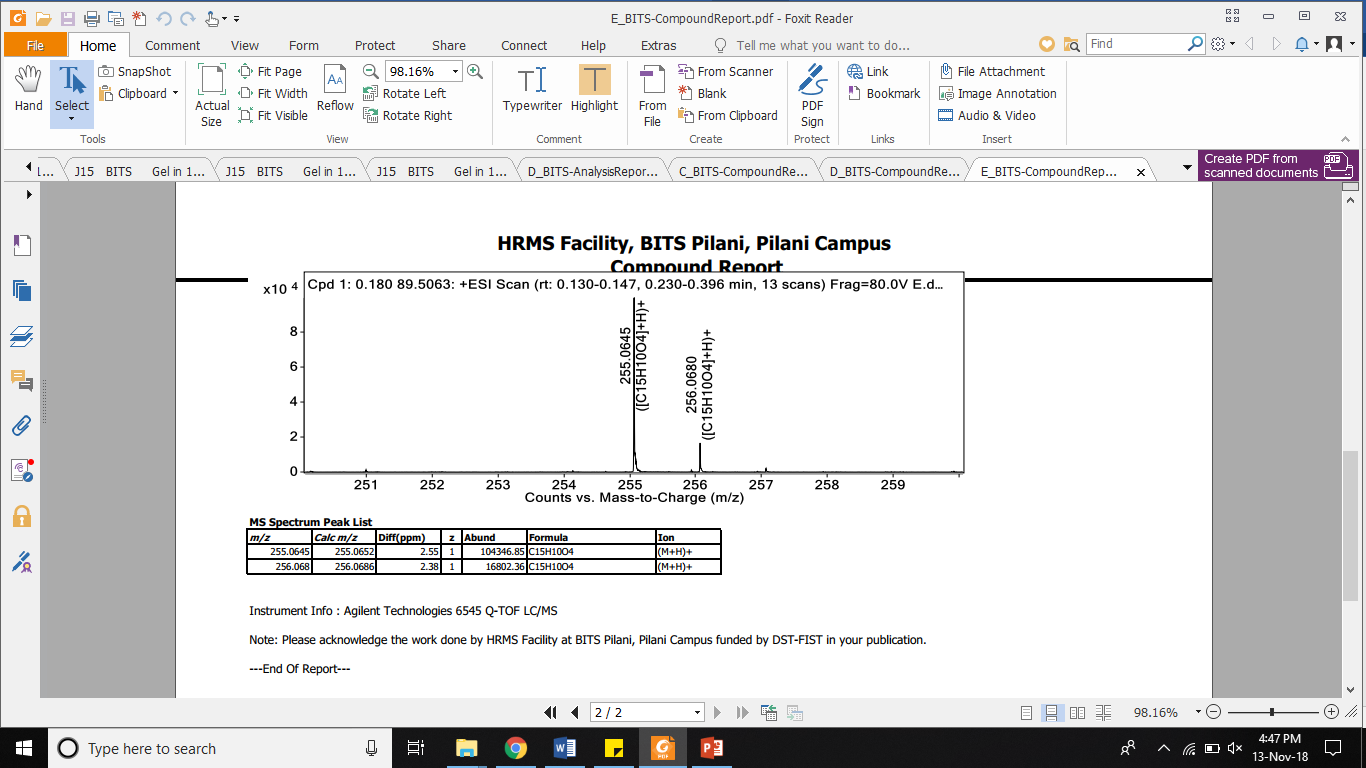


**Figure S1 : Mass spectra of standard Daidzein. Molecular mass of Daidzein is 254.241**


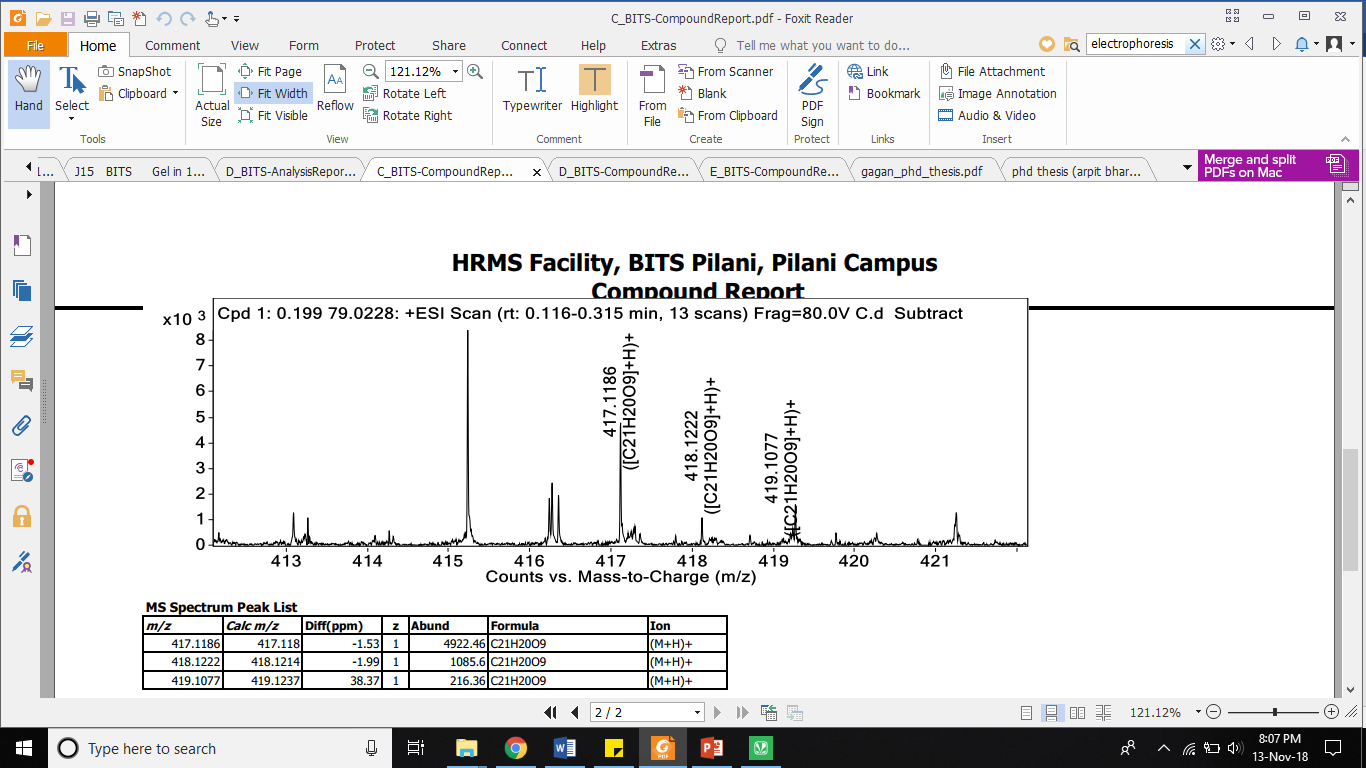


**Figure S2: Mass spectra of experimental control which consisted of Daidzin of molecular mass 416.38 as substrate.**


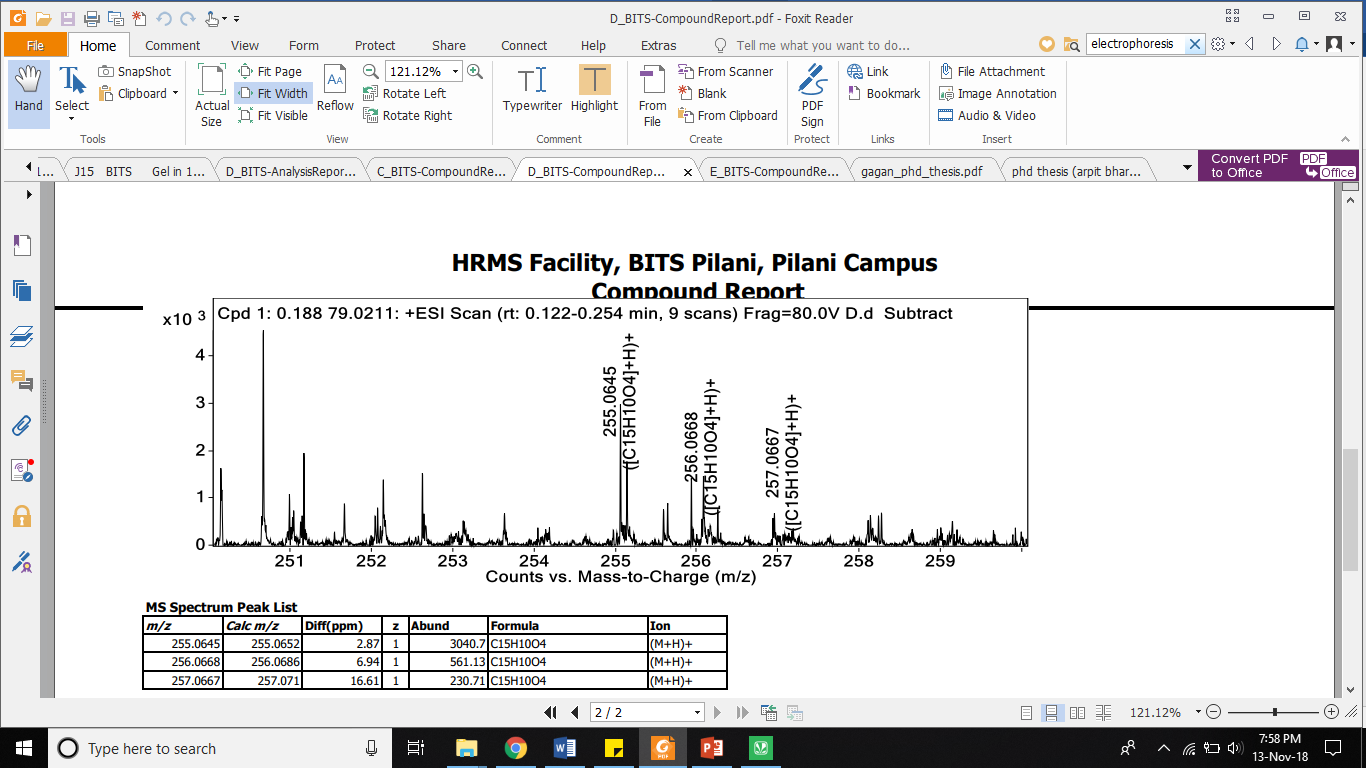


**Figure S3 : Mass spectra of experimental test which consisted of Daidzein as product released upon the enzymatic activity. The labelled peaks represent the parent peaks and further ionized peaks by different isotopes of hydrogen.**


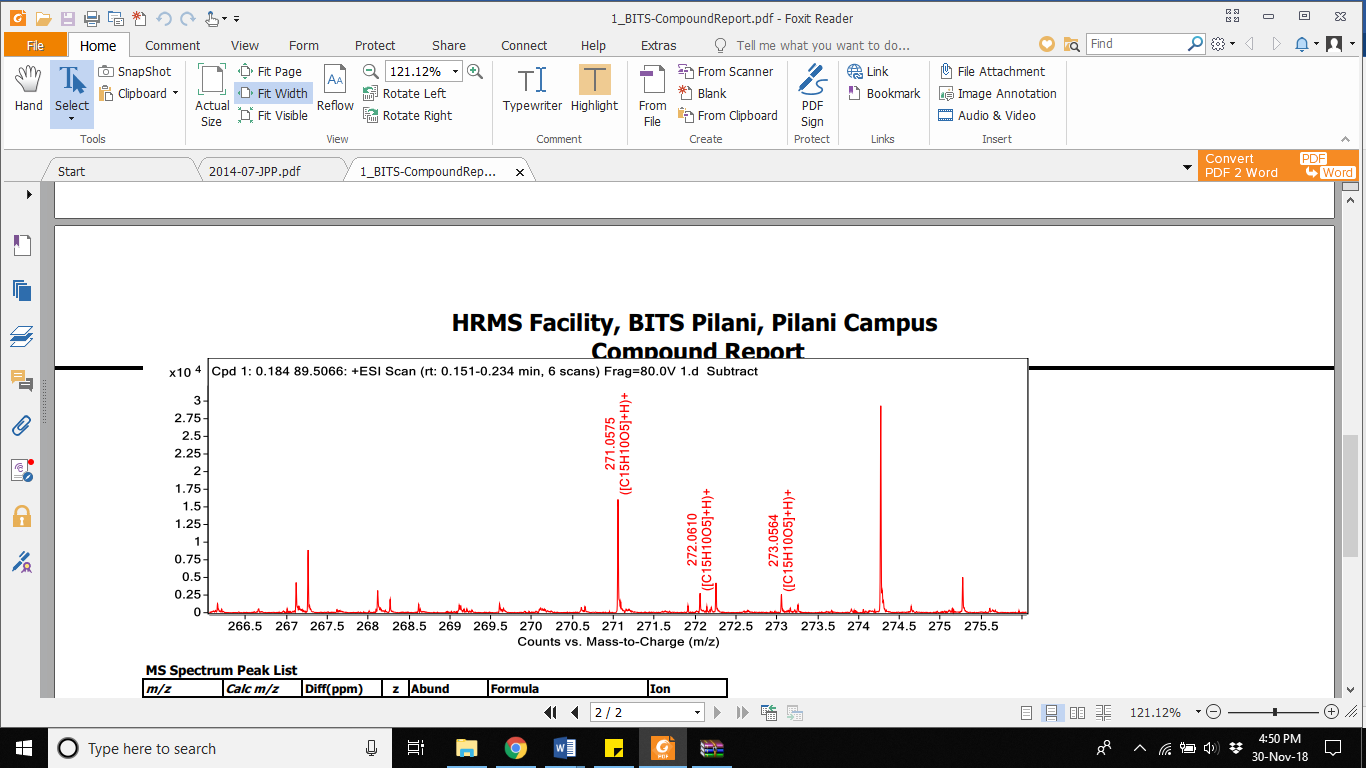


**Figure S4: Mass spectra of standard Genistein. Molecular mass of Genistein is 271.0575**

**Figure S5: Mass spectra of experimental control which consisted of Genistin of molecular mass 432.2343 as substrate**


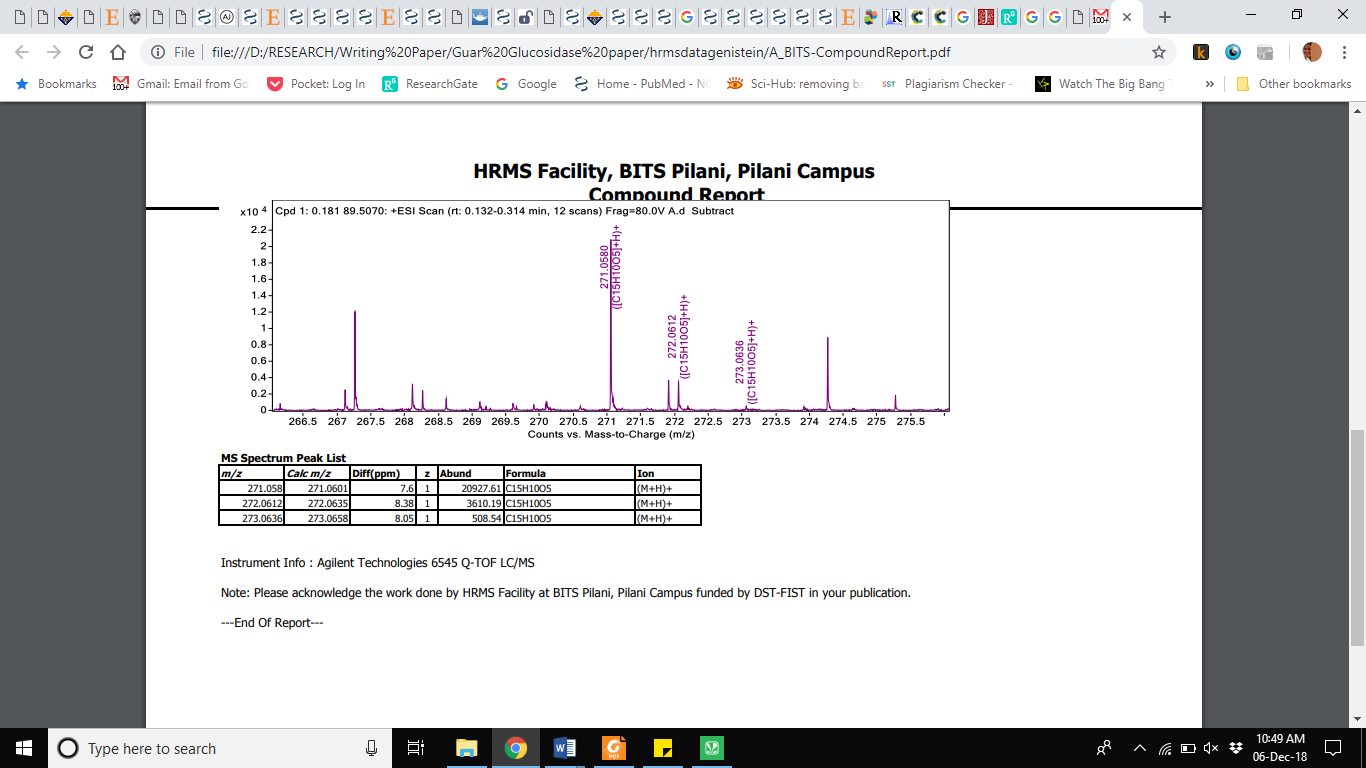


**Figure S6 : Mass spectra of experimental test which consisted of Genistin as substrate released aglycone Genistein upon enzymatic reaction. The m/z 271.0580 indicates the presence of Genistein**
